# Molecular identification and functional characterization of a transcription factor GeRAV1 from *Gelsemium elegans*

**DOI:** 10.1186/s12864-023-09919-9

**Published:** 2024-01-02

**Authors:** Tianzhen Cui, Shoujian Zang, Xinlu Sun, Jing Zhang, Yachun Su, Dongjiao Wang, Guran Wu, Ruiqi Chen, Youxiong Que, Qing Lin, Chuihuai You

**Affiliations:** 1 Key Laboratory of Sugarcane Biology and Genetic Breeding, Ministry of Agriculture and Rural Affairs, Key Laboratory of Genetics, Breeding and Multiple Utilization of Crops, Ministry of Education, College of Agriculture, Fujian Agriculture and Forestry University, Fuzhou, 350002 China; 2 College of Life Sciences, Fujian Agriculture and Forestry University, Fuzhou, 350002 China; 3 The Second People’s Hospital, Fujian University of Traditional Chinese Medicine, Fuzhou, Fujian 350003 China

**Keywords:** *Gelsemium elegans*, RAV transcription factor, Cold tolerance, Expression analysis, Prokaryotic expression

## Abstract

**Background:**

*Gelsemium elegans* is a traditional Chinese medicinal plant and temperature is one of the key factors affecting its growth. RAV (related to ABI3/VP1) transcription factor plays multiple roles in higher plants, including the regulation of plant growth, development, and stress response. However, RAV transcription factor in *G. elegans* has not been reported.

**Results:**

In this study, three novel *GeRAV* genes (*GeRAV1*-*GeRAV3*) were identified from the transcriptome of *G. elegans* under low temperature stress. Phylogenetic analysis showed that GeRAV1-GeRAV3 proteins were clustered into groups II, IV, and V, respectively. RNA-sequencing (RNA-seq) and real-time quantitative PCR (qRT-PCR) analyses indicated that the expression of *GeRAV1* and *GeRAV2* was increased in response to cold stress. Furthermore, the *GeRAV1* gene was successfully cloned from *G. elegans* leaf. It encoded a hydrophilic, unstable, and non-secretory protein that contained both AP2 and B3 domains. The amino acid sequence of GeRAV1 protein shared a high similarity of 81.97% with *Camptotheca acuminata* CaRAV. Subcellular localization and transcriptional self-activation experiments demonstrated that GeRAV1 was a nucleoprotein without self-activating activity. The *GeRAV1* gene was constitutively expressed in the leaves, stems, and roots of the *G. elegans*, with the highest expression levels in roots. In addition, the expression of the *GeRAV1* gene was rapidly up-regulated under abscisic acid (ABA), salicylic acid (SA), and methyl jasmonate (MeJA) stresses, suggesting that it may be involved in hormonal signaling pathways. Moreover, *GeRAV1* conferred improved cold and sodium chloride tolerance in *Escherichia coli* Rosetta cells.

**Conclusions:**

These findings provided a foundation for further understanding on the function and regulatory mechanism of the *GeRAV1* gene in response to low-temperature stress in *G. elegans.*

**Supplementary Information:**

The online version contains supplementary material available at 10.1186/s12864-023-09919-9.

## Background

Under low temperature stress, plants undergo significant alterations in morphology, physiology, molecular biology, and metabolism, leading to functional disruption and impairments in growth and development [1]. Transcription factors (TFs) are the major regulators in response to and coping with cold stress in plants [2]. The primary signal transduction pathways were activated in plants under low temperature stress depend on inducer of C-repeat binding factor expression/dehydration response elements binding protein (ICE-CBF/DREB) [3]. Besides, TFs like WRKY, leucine zipper (bZIP), v-myb avian myeloblastosis viral oncogene homolog (MYB), and NAC (NAM, ATAF1/2, CUC2) are capable of regulating gene expression and activating cold-regulated genes (CORs) [3]. The APETALA2/ethylene-responsive factor (AP2/ERF) family, which can be categorized into subfamilies including AP2, ERF, RAV, and Soloist, is one of the most extensive groups of TFs in plants [4]. Among them, RAV TFs, which not only contain AP2 domains but also have a B3 domain, are the specific class of TFs in higher plants, and are also classified as a B3 superfamily. The AP2 domain consisting of about 60 amino acids, which can recognize the CAACA motif, is located at the N-terminus of the RAV protein [4, 5]. Conversely, the B3 domain consisting of about 110 amino acids, which can recognize the CACCTG motif, is located in the conserved region at the C-terminus of the RAV protein [6]. Interesting, the presence of both domains in RAV TFs can enhance the specificity and affinity of binding to targeted DNA for controlling gene expression [4, 5].

Not surprisingly, in the AP2/ERF family, RAV TFs containing both AP2 and B3 domains have received increasing attention. In 1999, two novel RAV TFs (RAV1 and RAV2) have been identified for the first time from *Arabidopsis thaliana* [5]. Subsequent study has proved that there are 13 RAV TFs in *A. thaliana*, six of which contain both AP2 and B3 domains, including AtRAV1 (At1g13260), AtTEM1 (At1g25560), AtTEM2 (At1g68840), At3g25730, At1g50680, and At1g51120 [6]. The RAV family genes involved in plant growth and development and response to various stresses have been successively reported [7]. Overexpression of *Zea mays ZmRAV1* can enhance salt and osmotic tolerance in transgenic *A. thaliana* [8]. The promoter region of the RAV subfamily contains cis-acting elements involving low temperature response (LTR), and can thus regulate gene expression under cold stress [9]. Early cold stress rapidly induces overexpression of the *BcRAV* gene in *Brassica campestris* and improves its cold resistance [10]. The *VaRAV1* gene in *Vitis amurensis* plays a significant role in cold stress by regulating related TFs and inducing the expression of related genes in the cell wall [11]. To sum up, one hand, RAV IFs can regulate plant seed development, root growth, flower opening, and leaf senescence [12–15]. On the other hand, RAV IFs play important roles in biotic and abiotic stresses in plants, including disease, cold, drought, and salt resistance [8, 10, 16].

*Gelsemium elegans* (Gardner and Champ.) Benth., the medicinal vine plant commonly known as Gelsemium or “heartbroken grass”, was first recorded in the traditional Chinese medicinal book “Shennong Materia Media”. Although the entire plant of *G. elegans* is highly toxic, it has a high medicinal value [17]. The effective active components of *G. elegans* were indole alkaloids. Over a hundred different types of monoterpenoid indole alkaloids have been extracted from the roots [18], stems, leaves [19], fruits [20], and other organs of *G. elegans*, which provide a valuable reference for the development of new drugs [21, 22]. Researches have shown that the alkaloids in *G. elegans* are pharmacologically active in pain relief [23], anti-inflammatory effects [24], immunomodulatory [25], and therapeutic for depression and anxiety [26]. Surprisingly, nearly half of the common drugs come from natural products, especially alkaloids, making alkaloid-rich medicinal plants the center of attention [27]. *G. elegans* mainly grows in Southeast Asia and southern China [28]. Low temperature can affect the growth, metabolite biosynthesis and gene expression of medicinal plants, such as *Angelica sinensis* [29], *Dysosma versipellis* (Hance) M. Chang [30], and *Podophyllum hexandrum* Royle [31]. Similary, *G. elegans* cannot tolerate low temperatures during its growth. Prolonged exposure to low temperatures leads to morphological and physiological changes in *G. elegans*, which may affect the content and activity of its active ingredients [32].

Currently, there is still no report on the role of RAV TFs from *G. elegans* in cold tolerance. In this study, three *RAV* subfamily genes were identified and analyzed from the *G. elegans* transcriptome database constructed by our research group under low temperature stress (unpublished). Based on the transcriptome data and qRT-PCR analysis, one *G. elegans GeRAV1* gene significantly induced by 4°C low temperature was successfully cloned. Besides, the sequence characteristics, protein properties, structural domains, phylogenetic tree, subcellular localization, and self-activation activity of the *GeRAV1* gene were analyzed. Its expression in different *G. elegans* tissues and hormone treatments was detected by qRT-PCR technique. Moreover, the tolerance of *GeRAV1* to abiotic stress was investigated using prokaryotic expression systems. The findings of this study should serve as a basis for further exploration of the function and regulatory mechanism of RAV TFs in the response of *G. elegans* to low-temperature stress.

## Results

### Identification and sequence analysis of *G. elegans RAV* subfamily genes

**Fig. 1:**
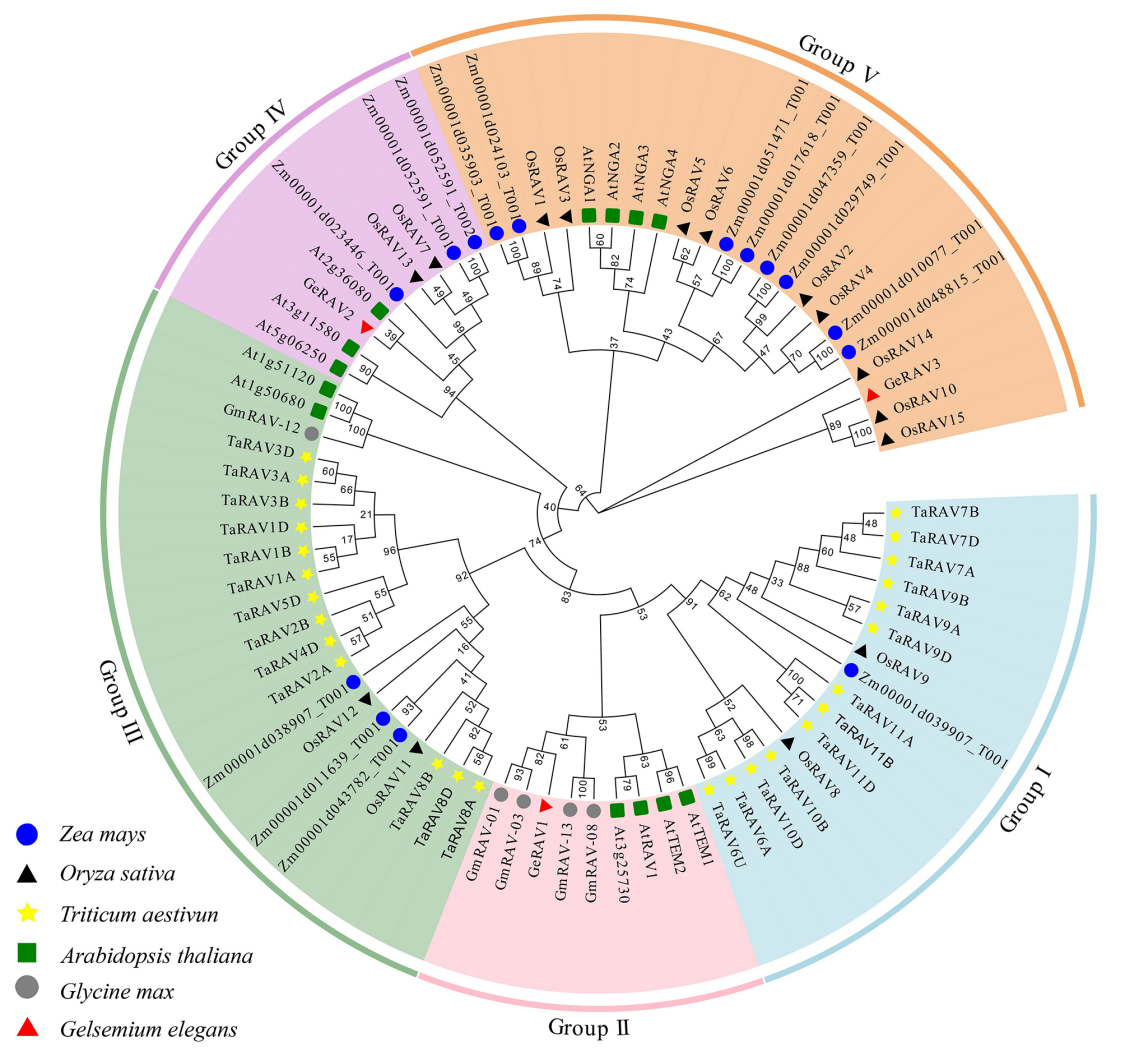
Phylogenetic tree of GeRAVs and other plant RAV proteins. The RAVs used in the phylogenetic tree analysis were from three dicot species (*Gelsemium elegans*, *Glycine max*, and *Arabidopsis thaliana*) and three monocot species (*Triticum aestivum*, *Oryza sativa*, and *Zea mays*) [33–35]. The GenBank accession numbers of all RAV homologous proteins were listed in Table S5. Groups I-V were marked in blue, pink, green, purple, and orange, respectively

**Fig. 2:**
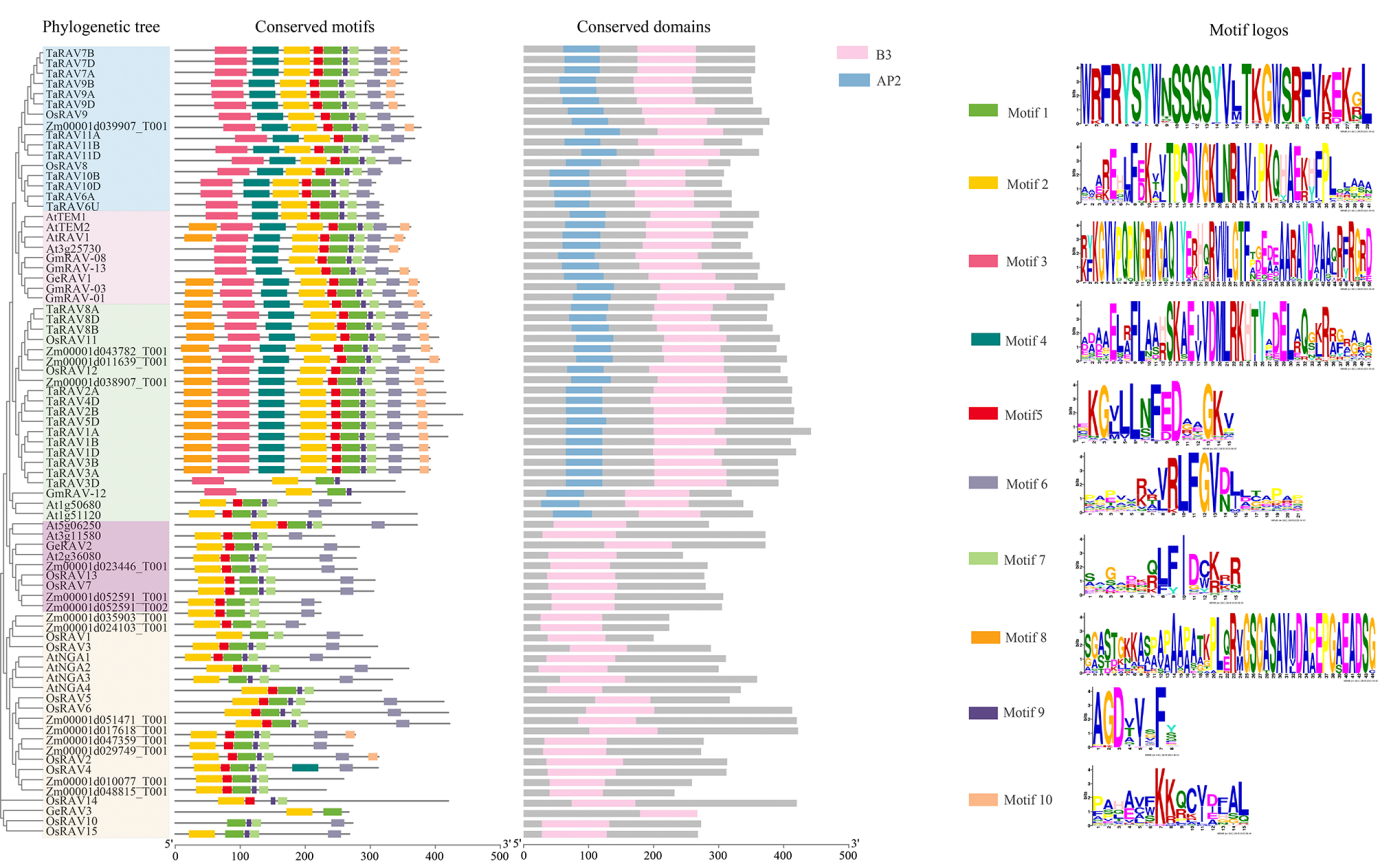
Phylogenetic tree, conserved motif, and conserved domain analyses of GeRAV1 and other species RAVs. Phylogenetic tree: the phylogenetic tree was the same as the evolutionary tree in Fig. 1. Conserved motif: the 10 conserved motifs corresponded to ten boxes of different colors. Conserved domains: B3 domains and AP2 domains were shown as pink and blue boxes, respectively. Motif logos: ten motif logo patterns and sequences were shown in Table S6

Three *GeRAV* genes (*GeRAV1*, c23896.graph_c0; *GeRAV2*, c34018.graph_c1; and *GeRAV3*, Gec35851.graph_c0) were identified from the transcriptome of *G. elegans* under 4°C low temperature stress which was previously constructed by our research group. According to those reports on the whole-genome analysis of the *RAV* gene family [33–35], a phylogenetic tree was constructed using the protein sequences of the GeRAV proteins and RAVs from two dicot plants (*A. thaliana* and *Glycine max*) and three monocot plants (*Z. mays*, *Oryza sativa*, and *Triticum aestivum*) (Fig. 1). A total of 77 RAV proteins were divided into five groups (Group I-V). Interestingly, GeRAV1 was clustered into Group II with proteins containing AP2 and B3 domains, and the other two GeRAVs (GeRAV2 and GeRAV3) were clustered into two respective groups (Group IV and Group V) containing only B3 domains. A total of ten conserved motifs were predicted for RAV proteins using the MEME website and 37.7% of RAV proteins contained more than nine motifs (Fig. 2). From the N-terminus to the C-terminus, all 77 RAV proteins contained motifs 3, 2, and 1, suggesting that they were relatively conserved in *RAV* genes evolution. Besides, in terms of conserved domains, all RAV proteins have B3 domains at the C-terminus, but do not always contain both AP2 and B3 domains (Fig. 2). In addition, the proteins in the groups I, II, and III had both AP2 and B3 domains (Fig. 2).

### The expression levels of *GeRAV* genes in response to low temperature stress

**Fig. 3:**
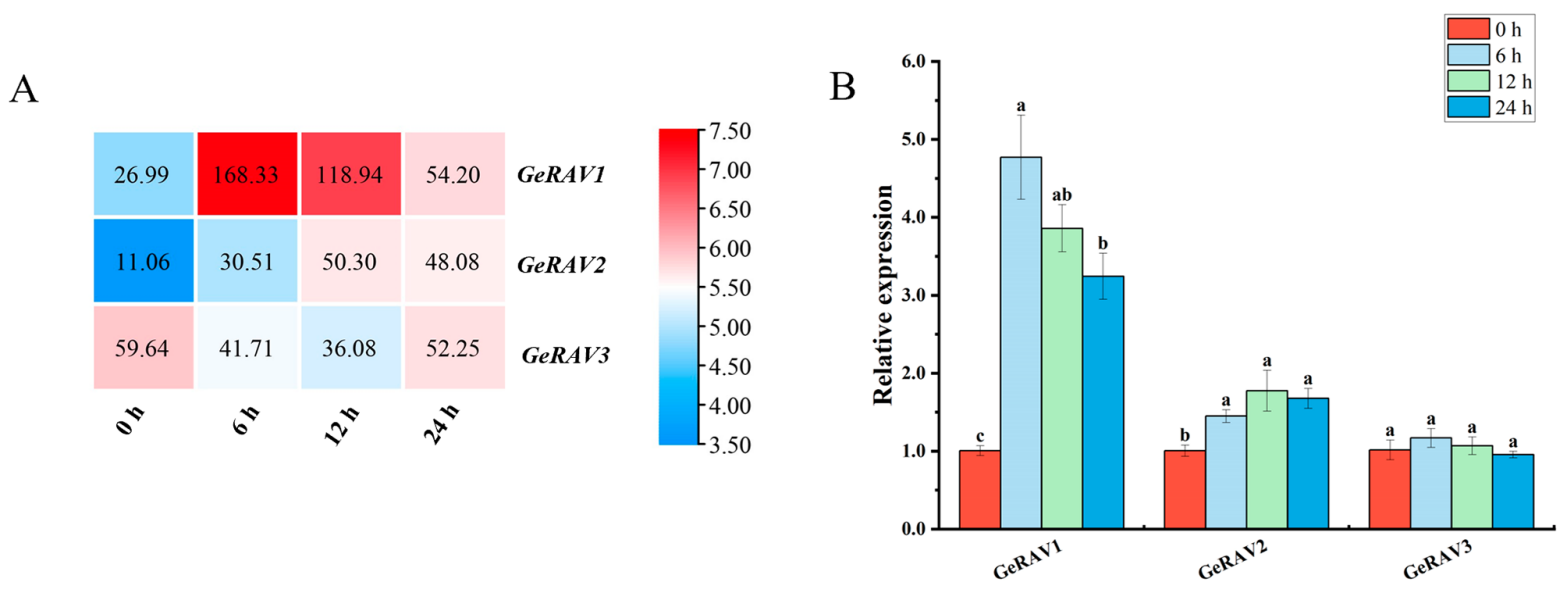
The expression of the *GeRAV* genes under 4°C low temperature treatment based on transcriptomic data **(A)** and qRT-PCR analysis **(B)**. In Fig. A, the number of fragments per kilobase of transcript per million mapped reads (FPKM) in the boxes represented the expression levels of *GeRAVs*. The color bar represented the normalized values (Log_2_ FPKM). In Fig. B, *GeCUL* was used as the reference gene. Different lowercase letters on the bars indicated significant differences, determined by Duncan’s new multiple range test (*P* < 0.05). Data points represented mean ± standard error (*n* = 3)

The expression profiles of the three *GeRAV* genes in *G. elegans* were analyzed under 4°C cold stress based on our transcriptomic data. The results showed that the expression of *GeRAV1* and *GeRAV2* were up-regulated under 4°C cold stress, whereas *GeRAV3* was down-regulated. *GeRAV1* exhibiting the highest expression profiles at 6 and 12 h (Fig. 3A). The qRT-PCR validation also showed that, under cold stress, the expression of *GeRAV1* gene maintained at a significant up-regulated level at 6, 12 and 24 h, which was about 4.75, 3.84 and 3.23 times higher than the control, respectively (Fig. 3B). Similarly, the expression of *GeRAV2* was also increased from 6 to 24 h, which was about 1.44, 1.77 and 1.67 times higher than the control, respectively (Fig. 3B). However, there was no significant change in the expression levels of the *GeRAV3* gene (Fig. 3B). These findings indicated that the *GeRAV1* and *GeRAV2* genes were induced by cold stress with up-regulated expression.

### Cloning and bioinformatics analysis of *GeRAV1*

As the *GeRAV1* gene was significantly up-regulated by low temperature stress (Fig. 3), its cDNA sequence (1272 bp) was amplified by RT-PCR using the RNA extracted from the leaves of *G. elegans* as a template. The *GeRAV1* gene contained an open reading frame (ORF) of 1083 bp, encoding a protein of 360 amino acids (Fig. S1) with a relative molecular weight of 40 kDa. The theoretical isoelectric point, the average hydrophilicity, and the instability coefficient of the GeRAV1 protein was 9.12, -0.554, and 40.03, respectively, suggesting that it was an unstable and hydrophilic protein. The absence of a signaling peptide or transmembrane region in the GeRAV1 protein indicated that it was a non-secretory protein. Secondary structure prediction results showed that the GeRAV1 protein was mainly composed of a random coil (50.28%), alpha helix (23.89%), extended chain (19.72%), and beta-turn (6.11%) (Fig. S2).

**Fig. 4:**
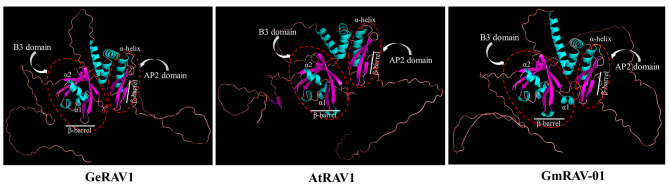
Predicted tertiary structures of *Gelsemium elegans* GeRAV1, *Arabidopsis thaliana* AtRAV1, and *Glycine max* GmRAV-01 proteins. The positions of the AP2 domain and the B3 domain were respectively outlined in red dotted lines. The AP2 domain included an α-helix and a β-barrel protein, while the B3 domain included two α-helices (α1 and α2) and a β-barrel protein

Comparative analysis of the predicted tertiary structures revealed that the GeRAV1 protein of *G. elegans* was highly similar to the RAV1 proteins of *A. thaliana* (At1g13260) and *G. max* (Glyma.01G22260). These three proteins contained an AP2 domain that consisted of an α-helix and a β-barrel protein, and the β-barrel protein was located parallel to the α-helix. In addition, the B3 domain of GeRAV1 contained two α-helices and one β-barrel protein, where the α-helices protruded from both ends of the β-barrel protein (Fig. 4). The amino acid sequence similarity of GeRAV1 with *Camptotheca acuminata* CaRAV (QNI23763.1), *Olea europaea* OeRAV1 (CAA2976529.1), *Vitis vinifera* VvRAV1 (XP_002281709.2), *Carya illinoinensis* CiRAV1-like (XP_042963336.1), *G. max* GmRAV-03 (Glyma.02G11060), and *A. thaliana* AtRAV1 was 81.97%, 79.95%, 74.55%, 76.55%, 59.31%, and 60.50%, respectively (Fig. S3). Notably, their amino acid sequence of the AP2 and B3 domains were highly conserved. The amino acid sequence of GeRAV1 showed certain conservation, with the N-terminal AP2 domain containing three β-strands (forming a β-barrel protein) and one α-helix, and the C-terminal B3 domain containing seven β-strands (forming a β-barrel protein) and two α-helices (α1 and α2) (Fig. S3). Furthermore, the predicted nuclear localization sequence (NLS) of the GeRAV1 protein was located at the C-terminus (Fig. S3).

### Relative expression of *GeRAV1* gene in *G. elegans* tissues and under hormonal stress

**Fig. 5:**
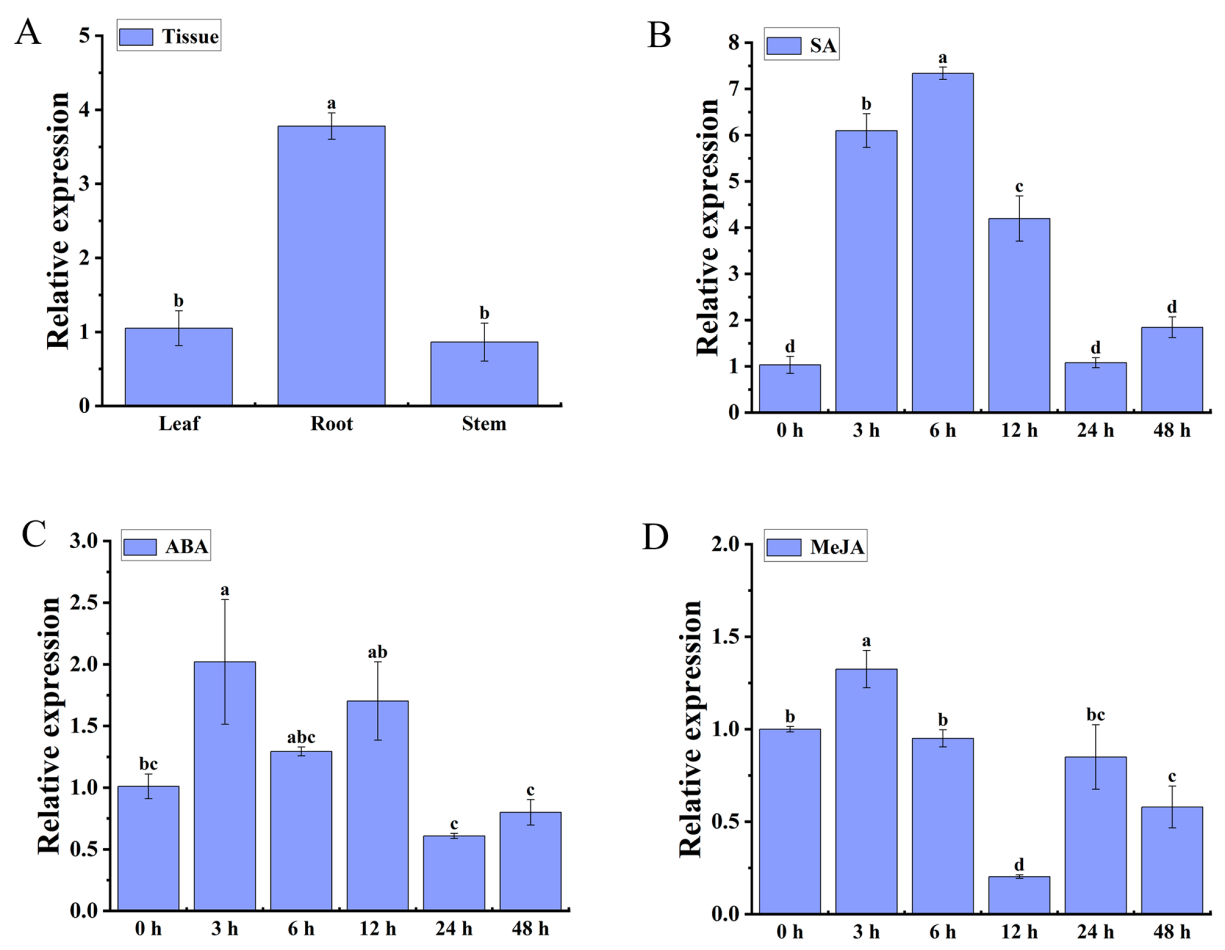
Relative expression analysis of the *GeRAV1* gene in different *Gelsemium elegans* tissues **(A)** and response to SA **(B)**, ABA **(C)**, and MeJA **(D)** stresses by qRT-PCR. *GeCUL* was used as the reference gene. Different lowercase letters on the bars indicated a significant difference, determined by Duncan’s new multiple range test (*P* < 0.05). Data points represented mean ± standard error (*n* = 3)

Expression levels of *GeRAV1* in the tissues of *G. elegans* were determined using the qRT-PCR technique. The results showed that the *GeRAV1* gene was constitutively expressed in different tissues of *G. elegans*, with the highest expression levels in the roots, which was approximately 3.6 and 4.38 times higher than those in the leaves and stems, respectively (Fig. 5A). In addition, the expression of *GeRAV1* was induced by treatments with salicylic acid (SA), abscisic acid (ABA), and methyl jasmonate (MeJA) (Fig. 5B, C, D). *GeRAV1* reached peak expression levels at 6 h under SA stress and at 3 h under both ABA and MeJA stimulus.

### GeRAV1 was localized to the nucleus

**Fig. 6:**
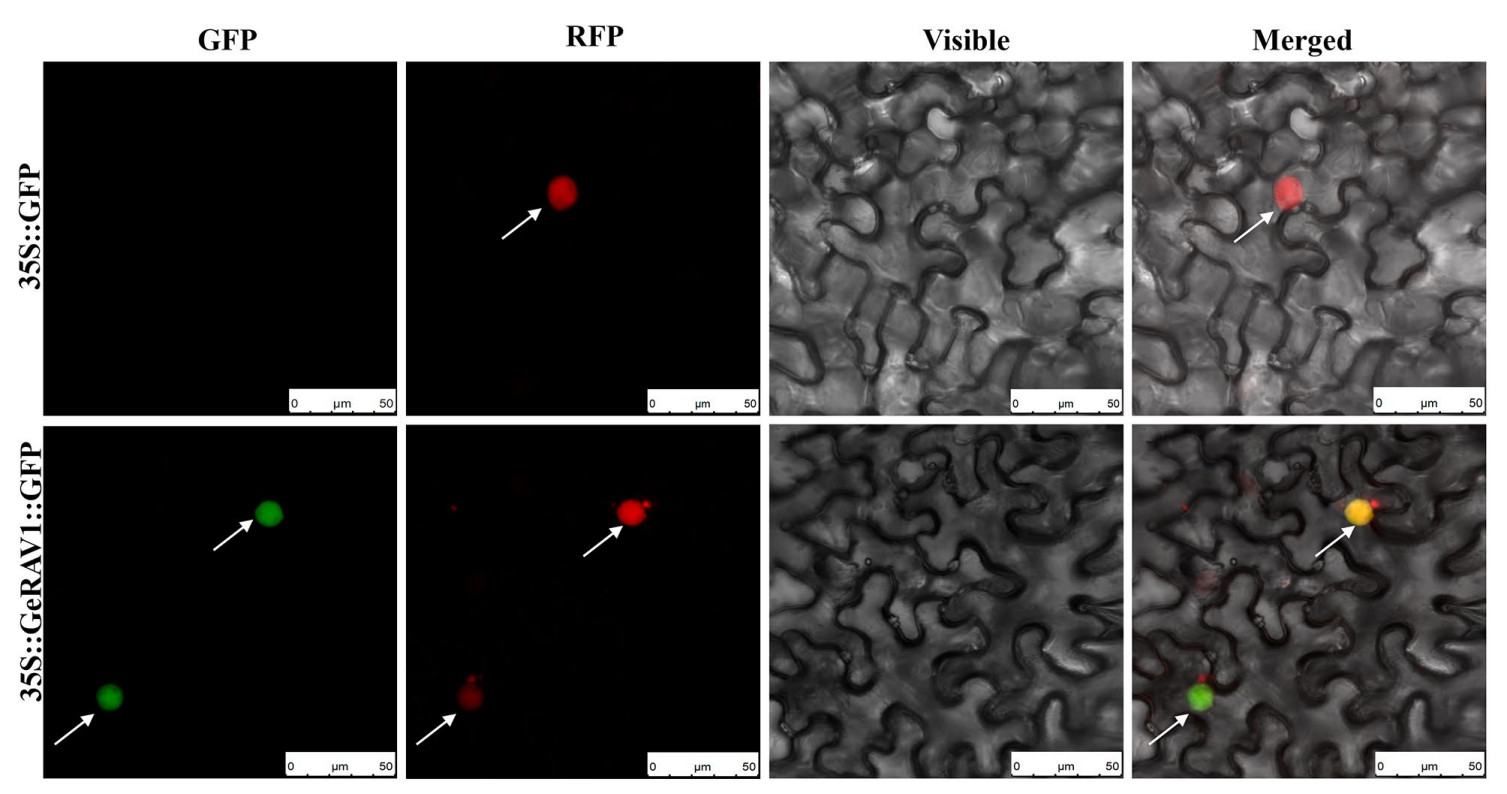
Subcellular localization of the GeRAV1 protein in *Nicotiana benthamiana*. The epidermal cells of *N. benthamiana* leaves were used for imaging analysis of bright field, green fluorescence (GFP), red fluorescence (RFP), and merged images of red and green fluorescence. White arrows indicated nucleus. Scale bar = 50 μm. 35S::GFP: *Agrobacterium* strain carrying empty vector pFASTR05-*GFP*. 35S::GeRAV1::GFP: *Agrobacterium* strain carrying recombinant vector pFAST-R05-*GeRAV1*-*GFP*. pFAST-R05 expresses a gene fused to a GFP, which followed the *ccdB* gene (with a stop codon). NbH2B (histone H2B)-RFP was used to indicate the nucleus

Subcellular localization of the GeRAV1 protein in the nucleus was predicted using WoLF PSORT and Plant-mPLoc online servers. To confirm this prediction, 35S::GeRAV1::GFP was used as an experimental group and 35S::GFP was used as a control group by *Agrobacterium*-mediated injections in *Nicotiana benthamiana* leaves. Confocal microscopy did not observe fluorescence signals in the control leaf cells, while the experimental group showed green fluorescence in the nucleus of leaf cells. Additionally, NbH2B (histone H2B) displayed red fluorescence in the nucleus of leaf cells. The overlap of green and red fluorescence resulted in yellow fluorescence, providing further evidence for the subcellular localization of the GeRAV1 protein in the nucleus (Fig. 6).

### *GeRAV1* had no self-activating activity

**Fig. 7:**
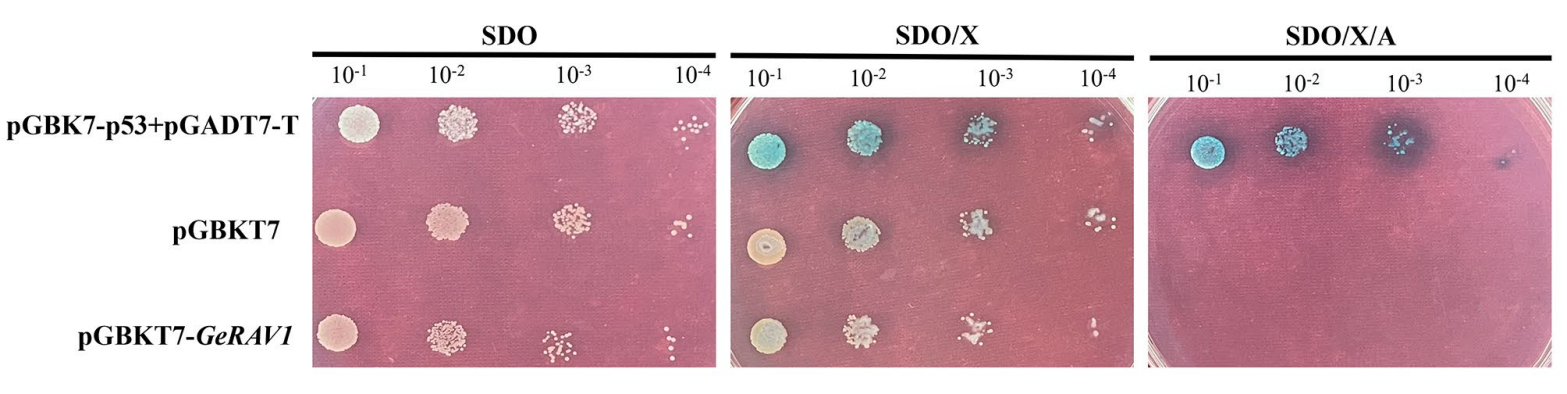
Transcriptional self-activation status of the GeRAV1 protein in yeast. SDO: Synthetic defined medium without tryptophan; SDO/X: synthetic defined medium without tryptophan supplemented with X-α-Gal; SDO/X/A: synthetic defined medium without tryptophan supplemented with X-α-Gal and aureobasidin A. pGBKT7-p53 + pGADT7-T: Positive control; pGBKT7: negative control

The presence of colonies on the tryptophan-deficient medium (SDO) plate of pGBKT7-*GeRAV1*, pGBKT7-p53 + pGADT7-T (positive control), and pGBKT7 (negative control) indicated the successful transformation of recombinant plasmids into Y2HGold yeast (Fig. 7). The growth of colonies showed a consistent trend with the gradient of diluted liquid culture, indicating that the GeRAV1 protein bound to GAL4-BD and expressed the missing tryptophan in the medium. Colonies of pGBKT7-*GeRAV* were observed on the SDO/X-α-gal (5-bromo-4-chloro-3-indole-α-dgalactoside) (SDO/X) plate, as well as positive and negative controls. However, upon the addition of X-α-gal, only the positive control displayed blue color due to the activation of the *MEL1* reporter gene, while the rest of the samples showed no color change, suggesting that the GeRAV1 protein had no self-activation activity. On the SDO/X/AbA (aureobasidin) (SDO/X/A) plate, only the positive control formed blue colonies, indicating activation of both *AUR1-C* and *MEL1* reporter genes. In contrast, no colonies were observed for the negative control and pGBKT7-*GeRAV1* (Fig. 7), further confirming the absence of transcriptional self-activation activity in the GeRAV1 protein.

### Stress tolerance assays of the *GeRAV1* gene in *Escherichia coli* Rosetta (DE3)

**Fig. 8:**
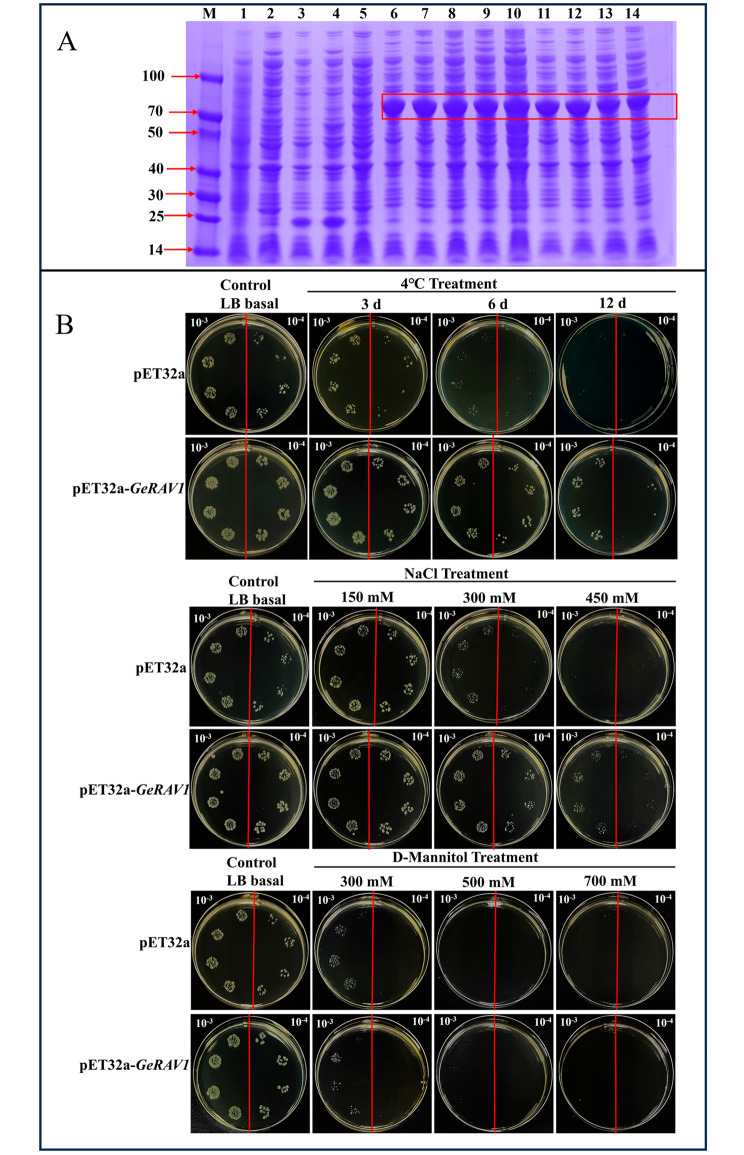
Expression analysis and plate stress validation of *GeRAV1* in *Escherichia coli* Rosetta (DE3) cells. **(A)** Induced expression of *GeRAV1* in *E. coli* Rosetta (DE3) cells. M: Protein marker; 1: blank control (uninduced *E. coli* Rosetta (DE3) cells); 2: blank control induced for 12 h; 3: control group (uninduced pET32a-Rosetta strain); 4: control group induced for 12 h; 5: uninduced recombinant strain (pET32a-*GeRAV1*-Rosetta strain); 6–10: recombinant strain induced at 37°C for 2, 4, 6, 8, and 12 h, respectively; 11–12: recombinant strain induced at 16°C for 2 and 12 h, respectively; 13–14: recombinant strain induced at 28°C for 2 and 12 h, respectively. The red boxes indicated the induced target protein. **(B)** Spot assay to verify the growth ability of pET32a-Rosetta cells and pET32a-*GeRAV1*-Rosetta cells on LB plates under 4°C, NaCl (sodium chloride), and mannitol stresses

The GeRAV1 protein has a predicted relative molecular weight of 40 kDa. pET32a expression vector contains a 6×His tag [36]. When recombinant cells (pET32a-*GeRAV1*-Rosetta) were successfully induced under specific conditions of 16°C, 28°C, 37°C, and 0.7 mM isopropyl β-D-thiogalactoside (IPTG), an obviously accumulated protein was observed above 40 kDa (Fig. 8A). As shown in Fig. 8B, the recombinant cells (pET32a-*GeRAV1*-Rosetta) had similar growth conditions as the control cells (pET32a-Rosetta) with mannitol supplement. However, the growth of recombinant cells (pET32a-*GeRAV1*-Rosetta) was significantly better than that of the control cells (pET32a-Rosetta) when treated with low temperature treatment at 4°C and sodium chloride (Fig. 8B). These results suggested that overexpression of *GeRAV1* in *E. coli* Rosetta could enhance its tolerance to cold and NaCl stresses.

## Discussion

Plants have evolved a molecular regulatory network and cold resistance mechanisms to cope with low temperature stress. It has been found that plant cells sense changes in temperature upon low-temperature stimulation, which triggers signaling receptors, such as Ca^2+^ permeation channels, receptor kinases, histidine kinases. [1, 37]. Subsequently, led by substances such as Ca^2+^, ABA, and reactive oxygen species, the low temperature signal was transmitted downward to activate TFs and regulate the expression of downstream CORs to enhance cold resistance [1, 37]. Within the AP2/ERF superfamily, the CBF/DREB TFs have been extensively and comprehensively studied in response to cold stress, as the CBF proteins can act on cis-acting elements and induce the expression of downstream CORs [1]. Recent research has identified RAV TFs belonging to the AP2/ERF family as cold-responsive TFs that may trigger regulatory pathways distinct from the CBF cold-responsive pathway [38]. Notably, RAV1 is a TF independent of the CBF pathway and plays a vital role in cold treatment and cold domestication [39]. Currently, RAV TFs respond positively to low temperature stress and have been cloned and studied in various plants such as *A. thaliana* [9], *O. sativa* [40], *Camellia sinensis* [41], *Galegae orientalis* [42], and *V. amurensis* [11].

### Sequences and phylogenetic characterization of *GeRAV1*

In this study, a *GeRAV1* gene with a significant response to low temperature stress was identified and characterized in the *G. elegans*. Tertiary structure prediction and multi-sequence alignments showed that the GeRAV1 protein exhibited similar structural features to AtRAV1 in *A. thaliana* (Fig. 4, Fig. S3), including the same number and the similar positions of α-helices and β-sheets in the AP2 and B3 domains, that is, as well as other reported RAVs containing the AP2 and B3 domains [6, 33, 43]. In addition, the AP2 and B3 domains showed a high degree of conservation between different amino acid sequences of RAVs from various plant species (Fig. S3), suggesting their important role in DNA binding specificity [44]. Interestingly, we also found the highest homology of 81.97% between *G. elegans* GeRAV1 and *C. acuminata* CaRAV (QNI23763.1) (Fig. S3). Therefore, it is hypothesized that there is a closer relationship between *G. elegans* and other genes in *C. acuminate*, which is conducive to our future research on alkaloid-related genes of *G. elegans*. Besides, GeRAV1 clustered closely with AtRAV1, At3g25730, AtTEM1, AtTEM2, GmRAV-03, GmRAV-01, GmRAV-08, and GmRAV-13 (Fig. 1), indicating that there is a closer evolutionary relationship between GeRAV1 and two dicotyledonous plants (*A. thaliana* and *G. max*) than the other three monocotyledonous plants (*Z. mays*, *O. sativa*, and *T. aestivum*). *GmRAV-03* has been reported to enhance NaCl and drought tolerance in transgenic *A. thaliana*, but is insensitive to exogenous ABA [33]. *A. thaliana AtRAV1* has been found to be involved in the cold response [9]. GeRAV1 belonged to the same group as GmRAV-03 and AtRAV1, with similar sequence type, number, and motif position (Fig. 2), indicating a similar biological function in the cold response. Previous studies have shown that the AP2 domain is a plant-specific domain involved in meristematic organization, flowering, seed development, and stress response [45]. The B3 domain binds to specific DNA and regulates plant growth, development, and hormone signaling [6, 46]. Considering that the GeRAV1 protein contained both AP2 and B3 domains, it is possible that *GeRAV1* may not only play a regulatory role in cold stress but also affect the growth and development of *G. elegans* and participate in certain hormone signaling pathways.

### *GeRAV1* gene was constitutively expressed in various tissues and responded positively to cold stress

Differential expression of the *GhRAV* gene has been observed in roots, stems, and leaves of *Gossypium hirsutum*, with a more significant increase in expression in roots and stems [47]. We found that the *GeRAV1* gene in *G. elegans* was expressed in root, stem, and leaf tissues, with a higher expression level in roots (Fig. 5A). *G. elegans* roots have higher levels and types of alkaloids than leaves and stems [28, 48], which was consistent with previous findings that over half of the extracted monoterpene indole alkaloids come from roots [22]. In the present study, we confirmed that the *GeRAV1* gene was highly expressed in *G. elegans* roots, does this imply a potential role in regulating the synthesis and accumulation of alkaloids in the roots of *G. elegans*?

As reported, the *RAV* genes exhibited a response to cold stress [38]. In maize seedlings, the *ZmRAV3* gene was significantly overexpressed after treatment at 4°C for 2 h [49]. The expression of the *BraRAV-14* gene in *Chinese cabbage* was up-regulated under low temperature at 4°C, peaking at 24 h [50]. The *RAV* family genes in *Brassica napus* showed up-regulated expression at four time points (2, 6, 12, and 24 h), with nearly half of the *RAV* genes reaching their highest expression levels at 6 h [10]. Based on the transcriptome data and qRT-PCR analysis, three *GeRAV* genes were differentially expressed in *G. elegans* at 4°C (Fig. 3). Interestingly, the expression levels of the *GeRAV1* gene were significantly up-regulated from 6 to 24 h under 4°C treatment. In addition, prokaryotic expression analysis showed that *GeRAV1* enhanced the tolerance of *E. coli* cells to low temperature (4°C) and NaCl treatments (Fig. 8B). These results indicating the potential role of the *GeRAV* gene in the response of *G. elegans* to low temperature and salt stresses. Similarly, previous studies have shown that *RAV* genes improved the tolerance of plants under NaCl and drought stresses [51, 52].

### Responses of *GeRAV1* to phytohormones stress

As one of the important signaling compounds, phytohormones can mediate plant response to abiotic stress [53]. The expression of the *GeRAV1* gene was significantly up-regulated at 3, 6 and 12 h under SA treatment and at 3 h under MeJA treatment (Fig. 5B, D), suggesting that the *GeRAV1* gene could be induced by SA and MeJA hormone stresses. Previous studies also showed that the expression of most *OsRAVs* in rice was up-regulated at 12 h under the treatments of MeJA and SA [35]. After challenging by ABA, the expression levels of *MtRAVs*, *GhRAV1*, and *GmRAV-03* were increased in *Medicago truncatula*, *Gossypium herbaceum*, and *G. max* [33, 54–56]. However, transgenic *A. thaliana* plants with the *MtRAVs*, *GhRAV1*, and *GmRAV-03* genes were insensitive to exogenous ABA, suggesting that these *RAV* genes might be participate in the ABA signaling pathway through an ABA-independent mechanism [33, 54–56]. In cucumber, *CsABI5* (ABA-associated marker gene) was significantly induced under ABA treatment and the transgenic *Arabidopsis* overexpressing *CsRAV1* was more tolerant to ABA, indicating that *CsRAV1* may directly regulate the expression of *CsABI5*, thereby participating in the ABA signaling pathway [57]. In this work, the *GeRAV1* gene was induced at 3, 6, and 12 h of ABA treatment (Fig. 5C). These results indicate that the *GeRAV1* gene of *G. elegans* may play a role in hormonal response. Further verification is needed to determine whether the *GeRAV1* gene is also involved in the ABA signaling pathway through an ABA-independent mechanism.

### Subcellular localization and transcriptional activation of GeRAV1 protein

In plants, RAV TFs were involved in regulating biosynthesis and metabolism. Zhao et al. demonstrated that the *NnRAV1* gene could interact with the *NnbHLH1* gene to regulate the synthesis and accumulation of alkaloids in *Nelumbo nucifera* [58]. In *Solanum tuberosum*, the *StRAV1* gene regulated anthocyanin accumulation in tubers by repressing the expression of key anthocyanin biosynthesis structural genes such as *StCHS*, *StANS*, and *St3’5’H* by suppressing their promoters [59]. *McRAV1* acted as an inhibitor of anthocyanin biosynthesis in *Malus crabapple* [60]. Similarly, in *M. esculenta*, the CAACA motif in the RAV TFs was common in the promoters of melatonin biosynthesis-related genes. *MeRAV1* and *MeRAV2*, as upstream TFs, directly activated three melatonin biosynthesis genes in cassava [61]. The *FaRAV1* gene directly bound and activated the promoters in the anthocyanin biosynthetic pathway that promoted anthocyanin accumulation in *Fragaria ananassa* [62]. Previous research demonstrated that anthocyanins confer a variety of colors to organs such as flowers and fruits, and play important roles in resisting stressors like low temperature and bright light [63]. RAV TFs in various plant species have been reported to function as nucleoproteins, such as *G. max* GmRAV-01 [64], *G. orientalis* GoRAV [42], *Manihot esculenta* MeRAV1/2 [65], *M. truncatula* MtRAV1/2/3 [54], *A. thaliana* AtRAV1 [12], *Betula platyphylla* BpRAV1 [66], *Capsicum annuume* CaRAV1 [16], *G. herbaceum* GhRAV1 [56], and *Z. mays* ZmRAV1 [8]. Similarly, the subcellular localization results indicated that the GeRAV1 was a nuclear-localized protein (Fig. 6). It is worth noting that ZmRAV1 in *Z. mays* and TaRAV1 in *T. aestivum* displayed transcriptional activity in yeast cells [8, 34], whereas NtRAV4 in *Nicotiana tabacum* did not [67]. In this study, the *GeRAV1* gene has no self-activation activity (Fig. 7), proving that it can be used as a bait, laying the foundation for mining functional genes that interact with it. This study will contribute to our further understanding of the biological function of the *G. elegans GeRAV1* gene. However, the role of RAV TFs in regulating biosynthesis and secondary metabolites in *G. elegans* still needs further investigation.

## Conclusion

In the present study, three novel *RAV* subfamily genes, whose encoding proteins were divided into three groups, were mined from the *G. elegans* transcriptome database under low temperature stress. Besides, the full-length *GeRAV1* gene, which was significantly induced by 4°C low temperature, was cloned and characterized. GeRAV1 was a nucleoprotein without self-activating activity. The expression of *GeRAV1* was observed in all the *G. elegans* tissues and the transcripts mainly accumulated in roots. Furthermore, *GeRAV1* mRNA expression was up-regulated under SA, MeJA, and ABA stimulus. In particularly, overexpression of *GeRAV1* could enhance the tolerance of *E. coli* Rosetta cells to low temperature and NaCl stresses, indicating that it is a potential candidate gene for genetic manipulation to improve low temperature and salt tolerance in *G. elegans*. These findings provide a basis for studying the biological functions and molecular regulatory mechanisms of RAV TFs in *G. elegans*.

## Materials and methods

### *G. elegans* materials and treatments

The *G. elegans* plants were derived from Yongding District, Fujian Province, China (at an altitude of 602 m, coordinates: 116.9°E, 24.4°N). These plant materials were identified by Professor Zhongyi Zhang from Fujian Agriculture and Forestry University as an evergreen woody vine plant belonging to the Loganiaceae family and Gelsemium genus. The roots, stems, and leaves of *G. elegans* were gathered and rapidly frozen in liquid nitrogen prior, and then stored in a -80°C freezer. Meanwhile, *G. elegans* seedlings were cultivated in a laboratory greenhouse under controlled conditions (25°C, 14 h of light, and 23°C,10 h of darkness). Subsequently, the *G. elegans* plants with uniform growth were selected and subjected to the following four experiments. Under 4°C low temperature treatment, leaf samples were collected at 0, 6, 12, and 24 h [68]. The leaves of *G. elegans* were subjected to a foliar spray of 100 µM MeJA, 5 mM SA, and 100 µM ABA [69, 70], with leaf samples being collected at 0, 3, 6, 12, 24, and 48 h [70, 71].

### Total RNA extraction and the first-strand cDNA synthesis

The *G. elegans* samples were ground to extract total RNA using the TRIzol method (Invitrogen, USA). According to the Hifair® cDNA Synthesis Kit (Yeasen, Shanghai, China), RNA from *G. elegans* leaves was reverse-transcribed into cDNA for subsequent gene cloning experiments. Based on the Hifair® cDNA Synthesis SuperMix kit (Yeasen, Shanghai, China), RNA of *G. elegans* tissues and *G. elegans* samples under different treatments was reverse-transcribed into cDNA and used as a template for qRT-PCR analysis.

### Mining *GeRAV* genes from *G. elegans*

Based on the transcriptome database of *G. elegans* under 4°C treatment, three *GeRAV* genes, namely *GeRAV1* (c23896.graph_c0), *GeRAV2* (c34018.graph_c1), and *GeRAV3* (c35851.graph_c0), were identified. The amino acid sequences of the RAV family members from *A. thaliana*, *O. sativa*, *Z. mays*, *G. max*, and *T. aestivum* were downloaded from TAIR, Phytozome, and Ensembl Plants databases [33–35] and were used for phylogenetic tree and conserved structural domains and motifs analyses with the three GeRAV proteins. Clustal X was used to perform multiple sequence comparisons. Evolutionary tree was constructed using MEGA X (Neighbor-Joining method, Bootstrap = 1000) [72]. EvolView was employed to beautify the evolutionary tree [73]. For conserved motifs analysis, the number of motifs was set to 10 with other parameters remaining at their default values by MEME website [74]. The prediction of conserved domains was carried out using NCBI-CDD (Table S1). TBtools was used to visualize the outcomes of the evolutionary tree, conserved domains, and conserved motifs [75].

### Cloning of *GeRAV1* gene and bioinformatics analysis

Based on the unigene sequence of the *GeRAV1* gene which has significant expression levels under low temperature stress, its cloning primers (Table S2, Table S3) were designed using Primer 5.0. The PCR products were ligated into the Blunt-Zero vector (Yeasen, Shanghai, China). The *GeRAV1* ORF sequence was analyzed using the ORF-finder program. The physicochemical properties, signal peptides, and transmembrane regions of the GeRAV1 protein were predicted using online software tools ProtParam, Net-Phos 3.1 server, SignalP 4.1 server, and TMHMM-2.0, respectively. The secondary structure of the protein was predicted using SOPMA. AlphaFold and PyMOL were used to predict and display the tertiary structure of the proteins, respectively [76]. Homologous amino acid sequences of GeRAV1 were searched using NCBI-blastp online website. Sequence comparison of GeRAV1 with other RAVs from the Dicot GenBank database was performed using DNAMAN6.0 software. The NLS was predicted using the online tool cNLS Mapper. The information of bioinformatics tools used was listed in Table S1.

### Expression patterns of the *GeRAV* genes in different tissues and under various stresses

The expression levels of the *G. elegans GeRAV* genes under low-temperature, MeJA, ABA, and SA treatments were examined using qRT-PCR technology. The details of designing quantitative primers for the *GeRAV* genes were shown in Table S3. The *GeCUL* gene of *G. elegans*, previously screened by our research group, was used as the reference gene (not yet published). qRT-PCR reaction system followed the instructions of the ChamQ Universal SYBR qPCR Master Mix (Nazyme, Nanjing, China). The qRT-PCR reaction was performed using the QuantStudio™ 6 Flex real-time fluorescence quantitative PCR system. The melting and standard curves of the *GeRAV* genes were shown in Table S4. Each sample was performed in triplicate and sterile water was used as a control. Relative expression levels of the *GeRAV1* genes were calculated using the 2^–ΔΔCT^ method [77] and data significance analysis was conducted using SPSS 19.0.0. Origin 2022 was used for data visualization and plotting.

### Subcellular localization

GeRAV1 subcellular localization was predicted using WoLF PSORT and Plant-mPLoc server online tools (Table S1). Based on the gateway technology system, the primers Subloc-*GeRAV1*-F/R (Table S2) were designed. The positive plasmid pEASY-Blunt Zero-*GeRAV1* obtained by cloning was served as a template for the BP reaction [78]. The product was then ligated into the entry vector pDONR207 to produce the positive plasmid pDONR207-*GeRAV1*. Subsequently, an LR reaction (Invitrogen, USA) was performed to connect it to the subcellular localization vector pFAST-R05, which expresses a gene fused to the green fluorescent protein gene (*GFP*) that follows the *ccdB* gene (with a stop codon) [79]. The positive recombinant plasmid pFAST-R05-*GeRAV1*-*GFP* (35S::GeRAV1::GFP) and the empty vector pFAST-R05-*GFP* (35S::GFP) were transformed into *A. tumefaciens* GV3101. After bacterial solution expansion and culture, the centrifuged culture was collected and washed twice with solution (10 mM magnesium chloride, 10 mM ethylmethylsulfone, pH = 5.0-5.4), then resuspended and adjusted OD_600_ ≈ 0.6, followed by the addition of 200 µM acetosyringone. Post 2–3 h of induction under dark conditions, the solution was injected into *N. benthamiana* leaves of 5–6 leaf ages. A bacterial solution containing NbH2B (histone H2B) was also injected into the leaves of *N. benthamiana* [80]. Subcellular localization of the GeRAV1 protein was observed using a Leica TCS SP8 confocal laser scanning microscope (Leica, Germany) after 2 days of incubation at 28°C and 16 h light/8 h dark.

### Transcriptional self-activation

The self-activation activity of GeRAV1 was investigated using the Matchmaker Gold yeast two-hybrid system (with *AUR1-C* and *MEL1* as reporter genes) [81]. First, the DNA-binding domain (BD) vector pGBKT7 was linearized by double digestion with *Eco*RI and *Bam*HI restriction enzymes. Primers pGBKT7-*GeRAV1*-F/R (Table S2), designed with specific restriction sites, were used for PCR amplification and gel purification. Then, the linearized vector and gel-purified target fragment were ligated for homologous recombination using the ClonExpress II One Step Cloning Kit (Vazyme, Nanjing, China). Finally, the recombinant plasmid pGBKT7-*GeRAV1*, BD-pGBKT7 vector (negative control), and hybridization vector plasmid pGBKT7-53 + pGADT7-T (positive control) were transformed into yeast Y2HGold competent cells (Weidi, Shanghai, China). After confirming the correctness of the cultures by PCR, the cultures were diluted to 1 × 10^–1^, 1 × 10^–2^, 1 × 10^–3^, and 1 × 10^–4^. Solid culture media including SDO, SDO/X, and SDO/X/A were prepared, and 7 µL of the diluted cultures were spotted onto the plates. All the plates were placed at 29°C for 2–3 d and photographed.

### Expression of *GeRAV1* gene in *E. coli* Rosetta (DE3) cells

According to the pET32a vector map, primers pET32a-*GeRAV1*-F/R (Table S2) were designed with restriction endonuclease sites *Bam*HI and *Hin*dIII to obtain the recombinant plasmid pET32a-*GeRAV1*. Subsequently, the recombinant plasmid pET32a-*GeRAV1* and the pET32a vector were transformed into *E. coli* Rosetta (DE3) cells (TIANGEN, Beijing, China). The *E. coli* Rosetta (DE3) cells containing pET32a-*GeRAV1*, pET32a vector, and no vector were each cultured in 20 mL centrifuge tubes, respectively, and their OD_600_ was adjusted between 0.6 and 0.7 and induced with 0.7 mM IPTG at 16°C, 28°C, and 37°C. All collected bacterial cultures were centrifuged and then loaded with a loading buffer. After boiling treatment at 100 °C, samples were subjected to sodium dodecyl sulfate-polyacrylamide gel electrophoresis for gel running. Following that, coomassie blue fast staining solution (Beyotime, Shanghai, China) was performed. The gel was then destained to observe the target bands. Finally, the FUSION FX.EDGE SPECTRA multi-color fluorescence & chemiluminescence (VILBER BIO IMAGING, Paris, France) was used for imaging.

The method of plate stress was used to investigate the response of *E. coli* Rosetta cells containing the *GeRAV1* gene under different stresses. When the bacterial cells (*E. coli* Rosetta (DE3) contains pET32a-*GeRAV1* and pET32a vectors, respectively) reached OD_600_ = 0.6, 0.7 mM IPTG was added to induce protein expression at 37°C. After 10 h of induction, the bacterial solution was adjusted to OD_600_ = 0.6 and then diluted again to 10^− 3^ and 10^− 4^. A total of 10 µL dilution was spotted on the corresponding plate, and then the stress tests of low temperature at 4°C (3, 6, 12 d), sodium chloride (150, 300, and 450 mM) and mannitol (300, 500, and 700 mM) were performed. Afterward, they were placed in a 37°C incubator overnight for the growth of bacterial colonies and then photographed [82].

### Electronic supplementary material

Below is the link to the electronic supplementary material.

Supplementary Material 1

Supplementary Material 2

## Data Availability

The data supporting the conclusions of this article are within the paper.

## Abbreviations

RAV: related to ABI3/VP1
ABA: abscisic acid
SA: salicylic acid
MeJA: methyl jasmonate
TFs: Transcription factors
ICE-CBF/DREB: C-repeat binding factor expression/dehydration response elements binding protein
bZIP: leucine zipper
MYB: v-myb avian myeloblastosis viral oncogene homolog
NAC: NAM,ATAF1/2,CUC2
CORs: cold-regulated genes
AP2/ERF: APETALA2/ethylene-responsive factor
LTR: cis-acting elements involving low temperature response
FPKM: fragments per kilobase of transcript per million mapped reads
NLS: nuclear localization sequence
RT-PCR: reverse transcription PCR
qRT-PCR: real-time quantitative PCR
RNA-seq: RNA-sequencing
IPTG: isopropyl β-D-thiogalactoside
NaCl: sodium chloride
*E. coli* Rosetta: *Escherichia coli* Rosetta
SDO: tryptophan-deficient medium
X-α-Gal: 5-bromo-4-chloro-3-indole-α-Dgalactoside
AbA: aureobasidin
GFP: green fluorescent protein
RFP: red fluorescence protein
