# Supplementary material for: Molecular identification and functional characterization of a transcription factor GeRAV1 from Gelsemium elegans
Source: BMC Genomics. 2024 Jan 2;25:22. doi: 10.1186/s12864-023-09919-9 (PMC10759518; doi:10.1186/s12864-023-09919-9)
Supplement: Supplementary file 1 — Supplementary Material 1 [file 12864_2023_9919_MOESM1_ESM.docx]

**Molecular identification and functional characterization of a transcription factor GeRAV1 from *Gelsemium elegans***

Tianzhen Cui ^1^, Shoujian Zang ^1^, Xinlu Sun ^1^, Jing Zhang ^1^, Yachun Su ^1^, Dongjiao Wang ^1^, Guran Wu ^1^, Ruiqi Chen ^3^, Youxiong Que ^1^, Qing Lin ^2,^*, Chuihuai You^1,3,^*

^1^ Key Laboratory of Sugarcane Biology and Genetic Breeding, Ministry of Agriculture and Rural Affairs, Key Laboratory of Genetics, Breeding and Multiple Utilization of Crops, Ministry of Education, College of Agriculture, Fujian Agriculture and Forestry University, Fuzhou 350002, China

^2^ The Second People’s Hospital Affiliated to Fujian University of Traditional Chinese Medicine, Fuzhou 350003, China

^3^ College of Life Sciences, Fujian Agriculture and Forestry University, Fuzhou 350002, Fujian, China

***Corresponding should be addressed to** [miranda100816@126.com](mailto:miranda100816@126.com) (Q. Lin) and [you123chui@163.com](mailto:you123chui@163.com) (C. You).

**The full postal address of the submitting author Youxiong Que is as follows:** Key Laboratory of Sugarcane Biology and Genetic Breeding, Ministry of Agriculture and Rural Affairs, College of Agriculture, Fujian Agriculture and Forestry University, Fuzhou, 350002, Fujian, China.

**Fig. S1** The cDNA sequence and amino acid sequence of *GeRAV1* gene. The red letters ATG and TAA represented the start codon and stop codon, respectively.


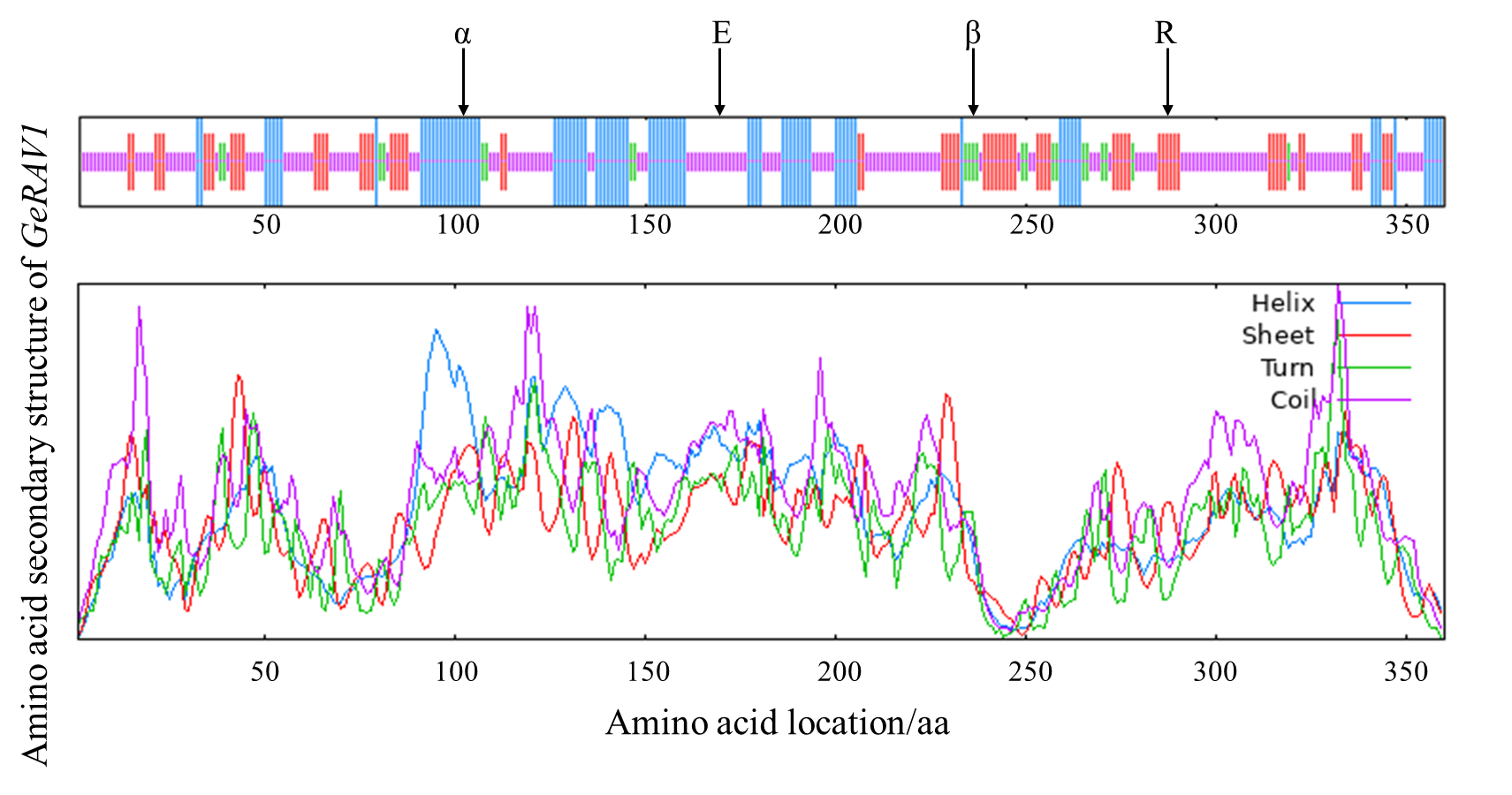


**Fig. S2** Prediction of the secondary structure of GeRAV1 protein. α: α-helix; E: extended chain; β: β-turn; R: disordered coil.


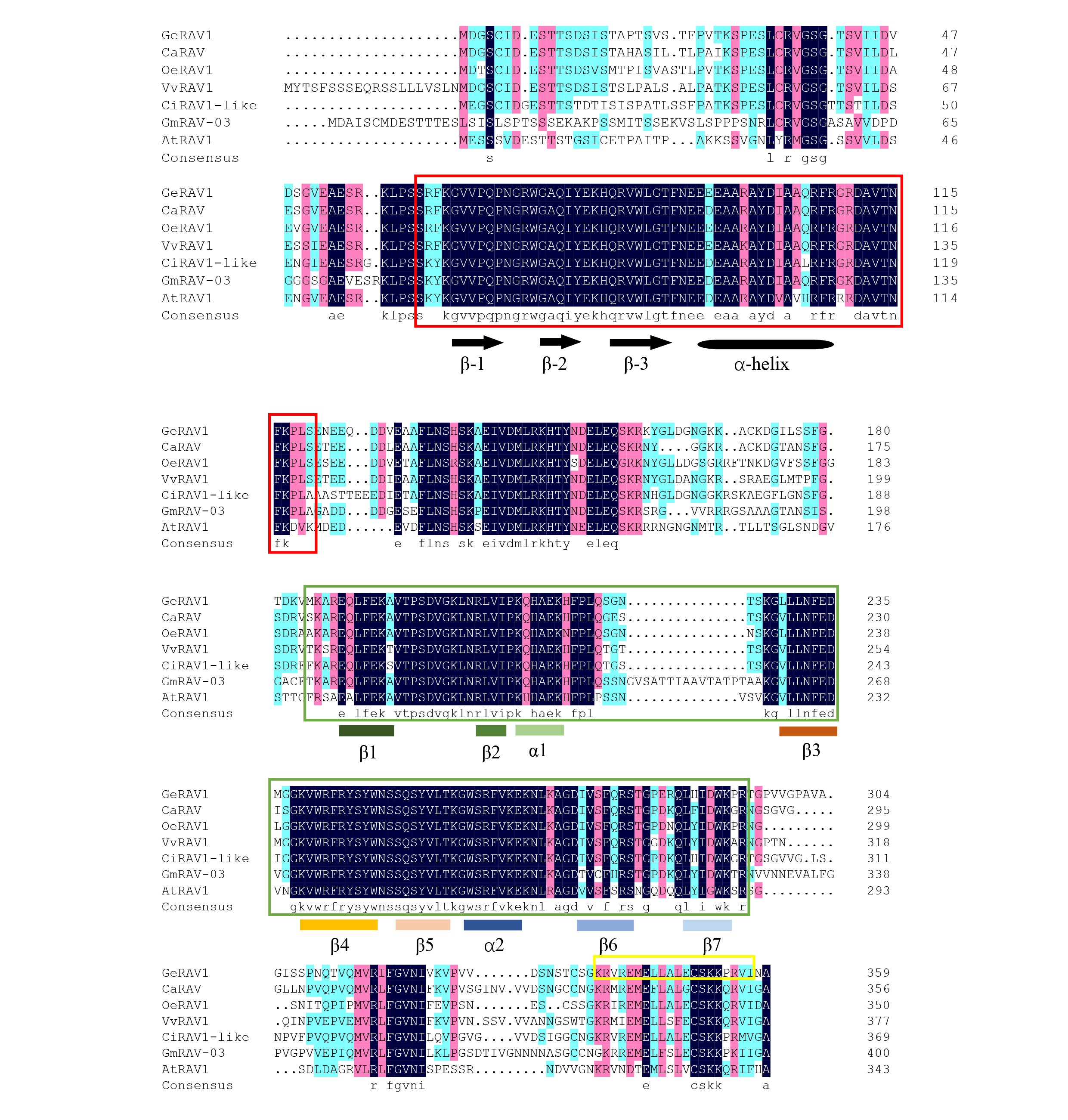


**Fig. S3** Protein sequence alignment of *Gelsemium elegans* GeRAV1 and RAVs from other plant species. Red box: AP2 domain; green box: B3 domain; yellow box: nuclear localization signal (NLS). The amino acid sequences of *Camptotheca* *acuminata* CaRAV (QNI23763.1), *Olea europaea* OeRAV1 (CAA2976529.1), *Vitis* *vinifera* VvRAV1 (XP_002281709.2), *Carya* *illinoinensis* CiRAV1-like (XP_042963336.1), *Glycine max* GmRAV-0 3 (Glyma.02G11060), and *Arabidopsis thaliana* RAV1 (At1g13260) were obtained from NCBI GenBank. The colors black, yellow, blue, and white represented the degree of homology in the alignment of amino acid residues at 100%, ≥75%, ≥50%, and <50%, respectively.
